# Structural Analysis of Crystalline *R*(+)-α-Lipoic Acid-α-cyclodextrin Complex Based on Microscopic and Spectroscopic Studies

**DOI:** 10.3390/ijms161024614

**Published:** 2015-10-16

**Authors:** Naoko Ikuta, Takatsugu Endo, Shota Hosomi, Keita Setou, Shiori Tanaka, Noriko Ogawa, Hiromitsu Yamamoto, Tomoyuki Mizukami, Shoji Arai, Masayuki Okuno, Kenji Takahashi, Keiji Terao, Seiichi Matsugo

**Affiliations:** 1 Graduate School of Medicine, Kobe University, Kobe 650-0017, Japan; E-Mails: naoko.ikuta@people.kobe-u.ac.jp (N.I.); keiji.terao@cyclochem.com (K.T.); 2 College of Science and Engineering, Kanazawa University, Kanazawa 920-1192, Japan; E-Mails: tkendo@staff.kanazawa-u.ac.jp (T.E.); hsmst14@gmail.com (S.H.); peridot@staff.kanazawa-u.ac.jp (T.M.); ultrasa@staff.kanazawa-u.ac.jp (S.A.); mokuno@staff.kanazawa-u.ac.jp (M.O.); ktkenji@staff.kanazawa-u.ac.jp (K.T.); 3 Department of Pharmaceutical Engineering, School of Pharmacy, Aichi Gakuin University, Nagoya 464-8650, Japan; E-Mails: tektites5@gmail.com (K.S.); yfd44023@nifty.com (S.T.); noriko30@dpc.agu.ac.jp (N.O.); hiromitu@dpc.agu.ac.jp (H.Y.); 4 CycloChem Bio Co., Ltd., Kobe 650-0047, Japan

**Keywords:** cyclodextrin, lipoic acid, ATR/FT-IR, microscopic Raman, solid-state NMR

## Abstract

*R*(+)-α-lipoic acid (RALA) is a naturally-occurring substance, and its protein-bound form plays significant role in the energy metabolism in the mitochondria. RALA is vulnerable to a variety of physical stimuli, including heat and UV light, which prompted us to study the stability of its complexes with cyclodextrins (CDs). In this study, we have prepared and purified a crystalline RALA-αCD complex and evaluated its properties in the solid state. The results of ^1^H NMR and PXRD analyses indicated that the crystalline RALA-αCD complex is a channel type complex with a molar ratio of 2:3 (RALA:α-CD). Attenuated total reflection/Fourier transform infrared analysis of the complex showed the shift of the C=O stretching vibration of RALA due to the formation of the RALA-αCD complex. Raman spectroscopic analysis revealed the significant weakness of the S–S and C–S stretching vibrations of RALA in the RALA-αCD complex implying that the dithiolane ring of RALA is almost enclosed in glucose ring of α-CD. Extent of this effect was dependent on the direction of the excitation laser to the hexagonal morphology of the crystal. Solid-state NMR analysis allowed for the chemical shift of the C=O peak to be precisely determined. These results suggested that RALA was positioned in the α-CD cavity with its 1,2-dithiolane ring orientated perpendicular to the plane of the α-CD ring.

## 1. Introduction

**Figure 1:**
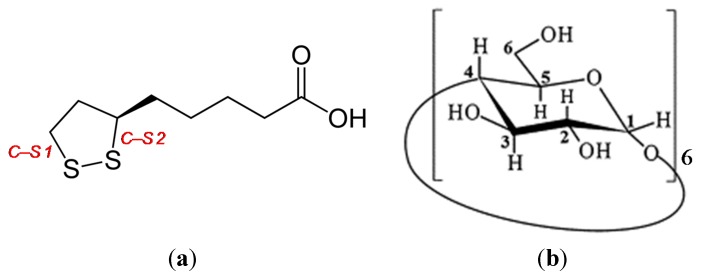
Chemical structures of (**a**) *R*(+)-α-lipoic acid (RALA) and (**b**) α-cyclodextrin.

Naturally-occurring cyclodextrins (CDs) are all cyclic, non-reducing oligosaccharides, consisted of α-1,4-linked glycopyranose units and are named as α-CD, β-CD, and γ-CD according to the number of glucose molecules of six (α-CD) (Figure 1), seven (β-CD), or eight (γ-CD). Compounds belonging to this structural class are cylindrical in shape. The hydrophilic hydroxyl groups of CDs sit on their outer surface, whilst the remaining hydrophobic portions of the molecule form different sized hydrophobic cavity (based on the CD ring size) at the center. CDs are consequently capable of forming host-guest complexes with water-insoluble substances, such as lipophilic substrates by completely or partially encapsulating these molecules in their cavity through a series of non-covalent interactions. Notably, these interactions can have a significant improvement for the non-water soluble and/or vulnerable guest molecules [1,2,3,4,5]. The structures of these host-guest complexes have attracted considerable interest from numerous researchers, and there have been several studies aimed at developing a better understanding of these complexes focusing on the relation of the binding constants and the ring size of the CD [6,7].

Most of these experiments were conducted in the liquid phase, and changes in the spectral properties upon complexation were measured by using NMR and UV-Vis spectroscopy. Unfortunately, however, these changes can only be observed for guest molecules that interact strongly with the CD host, meaning that it is not possible to study molecules with weak binding constants using these analytical techniques. There are many commercially-available CDs and their derivatives, and recent development of various experimental techniques makes it possible to measure these compounds and their interactions with guest molecules in the solid state accurately. Among such instruments, differential scanning calorimetry (DSC), scanning electron microscopy (SEM), and X-ray diffraction (XRD) analysis are the most popular ones. Despite these advances, the development of an alternative analytical technique to analyze the interactions present in the host and guest molecules with small binding constants is highly desired.

α-Lipoic acid (ALA), bearing both hydrophobic and hydrophilic groups, shows strong antioxidant activity in both aqueous and non-aqueous media (Figure 1). ALA does not have a strong affinity for CDs [8], which means that it can be difficult to accurately measure small changes in its spectral properties using UV or Fourier transform infrared (FT-IR) spectroscopy. Microscopic FT-IR is applied to pursue an accurate analysis. In terms of its structure, ALA has two C–S bonds and one S–S bond making up a five-membered 1,2-dithiolane ring, as well as a chiral center on its C_6_ carbon. This presence of a chiral center means that ALA can exist as two optically-active enantiomers, including *R*(+)-ALA (RALA) and *S*(−)-ALA (SALA), where RALA is the naturally-occurring enantiomer. We recently reported that RALA could be stabilized through the formation of a complex with CD to give RALA-CD. We also examined the physicochemical characteristics of a series of RALA-CD complexes by a usage of DSC, SEM, XRD and HPLC [9]. Nevertheless, it was not possible in this instance to clarify the nature of the interactions between the host and guest molecules of these complexes in the solid state [9]. Notably, spectroscopic analysis of the RALA-CD complexes using a combination of Raman and FT-IR analysis provided structural information that showed that the 1,2-dithiolane and carbonyl moieties of RALA were interacting with the CDs [10]. The purpose of the current study was to estimate the specific sites of the intermolecular interactions between the individual molecules in the RALA-CD complexes. In this regard, we initially tried to measure the RALA-CD complexes using solid-state NMR, but it was quite difficult to observe peaks that were suitable (*i.e*., sharp enough) for analysis. Actually, our preliminary solid-state NMR spectra of the RALA-CD complexes were too broad to be deconvoluted into distinctive peaks. This result indicated that the structures of the RALA-CD complexes are disordered. We subsequently attempted to purify the RALA-CD complexes using a supersaturation technique and subsequently obtained crystalline RALA-CD complexes. The focus of our study then turned towards the solid-state analysis of these crystalline complexes to investigate the effect of the complexation process on the S–S and C–S bonds of the 1,2-dithiolane ring of RALA. Herein, we report for the first time the preparation of crystalline RALA-αCD complexes and the examination of their formation using attenuated total reflectance (ATR)/FT-IR, microscopic-Raman with near-infrared excitation, and solid-state NMR analyses. The results give more detailed information on the arrangement of RALA molecules in the CD rings.

## 2. Results and Discussion

In this study, we used supersaturated aqueous solutions of non-crystalline RALA-αCD as raw materials to prepare a crystalline RALA-αCD complex, which was evaluated using various analytical techniques. The results of previous study indicated that the molar ratio of α-CD to RALA needed to be higher than 1:1 to allow for the formation of a RALA-αCD complex [9]. With this in mind, we used molar ratio of 1:2 and 2:3 ratio (RALA:α-CD) for the formation of the non-crystalline RALA-αCD complexes.

### 2.1. ^1^H-NMR Analysis

The ^1^H NMR spectrum of CD contains six clearly identifiable proton signals, including those of protons H_1_, H_2_, and H_4_, which are on the outer surface of CD; protons H_3_ and H_5_, which sit on the inner surface (or cavity) of the CD ring and are very important for studying the formation of interactions between CD and guest compounds; and H_6_ (*i.e*., CH_2_OH, Figure 1b), which is located on the minor edge cavity of CD [11].

The molar ratio of RALA to α-CD for the crystalline RALA-αCD complexes was calculated by ^1^H NMR spectroscopy based on the relative intensities of the proton signals belonging to the primary hydroxyl groups of α-CD and the carboxylic acid proton of RALA. The results of this analysis revealed that the molar ratio of α-CD to RALA in the crystalline RALA-αCD complexes was 3:2, even when the crystalline RALA-αCD complexes were prepared from non-crystalline RALA-αCD complexes with molar ratios of 1:2 or 2:3.

### 2.2. Scanning Electron Microscopy (SEM)

**Figure 2:**
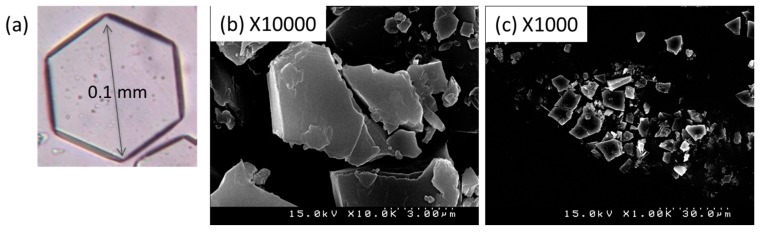
Optical micrograph of a single crystal of the crystalline RALA-αCD complex (**a**) and SEM images of the crystalline RALA-αCD complex with ×10,000 magnification (**b**) and ×1000 magnification (**c**).

Figure 2 shows an optical micrograph of a single crystal of the crystalline RALA-αCD complex, as well as SEM images of the same material. As shown in Figure 2a, the single crystal of the crystalline RALA-αCD complex was thin and hexagonal in shape. The SEM images of this material showed that its surface was smoother than that reported previously for the corresponding non-crystalline RALA-αCD complex [9]. Triplicate observations were carried out in different fields for each sample following the collection of images with magnifications of 500×, 1000×, and 10,000×. Figure 2b,c shows the images for magnifications of 10,000× and 1000×, respectively.

The particles making up the crystalline RALA-αCD complex were around 3–10 μm in size and appeared to be part of a larger hexagon. These results indicated that the morphological features of the crystalline RALA-αCD complexes were completely different from those of the non-crystalline RALA-αCD complex and that they packed in a hexagonal manner.

### 2.3. Powder X-ray Diffraction

**Figure 3:**
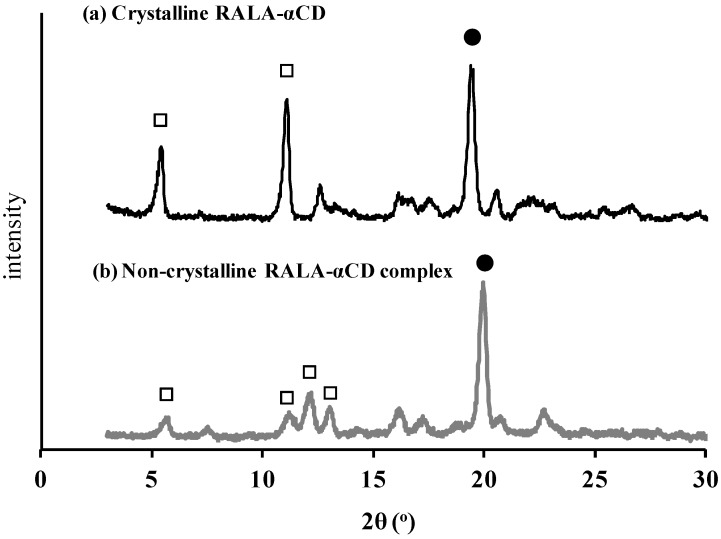
PXRD patterns of the crystalline (**a**) and non-crystalline (**b**) RALA-αCD complexes. Solid circle: peaks that indicate channel type structure. Hollow square: peaks changed through crystallization.

Powder X-ray diffraction (PXRD) is the commonly used method for the detection of CD complexation either in the powder and microcrystalline states [12]. The PXRD profiles of the crystalline and non-crystalline RALA-αCD complexes are shown in Figure 3.

As shown in Figure 3a, the PXRD pattern of the crystalline RALA-αCD complex gave three characteristic diffraction peaks at 2θ = 5.5°, 11°, and 19°, while that of the non-crystalline material gave one strong peak at 2θ = 20°. The peaks around 2θ = 20° (marked as filled circles in Figure 3) indicated that these complexes were channel type structure. The relative intensities of the peaks marked as square frame to the peaks around 2θ = 20° (marked as filled circles) in Figure 3 were got stronger in the crystalline RALA-αCD compared to the non-crystalline RALA-αCD complex and the peak number was reduced from the non-crystalline to the crystalline RALA-αCD complex. This result suggested that the crystalline RALA-αCD complex was purer than the non-crystalline material and that the structures had converged. The basic host structure of the crystalline RALA-αCD complex was similar to that of the typical channel type structure found in CD inclusion compounds [13,14,15].

Figure 3 shows that the PXRD patterns and relative peak intensities of the crystalline and non-crystalline RALA-αCD complexes were different, which therefore suggests that our supersaturation technique successfully facilitated the crystallization of the RALA-αCD complex.

### 2.4. Fourier Transform Infrared (FT-IR) Spectroscopy

**Figure 4:**
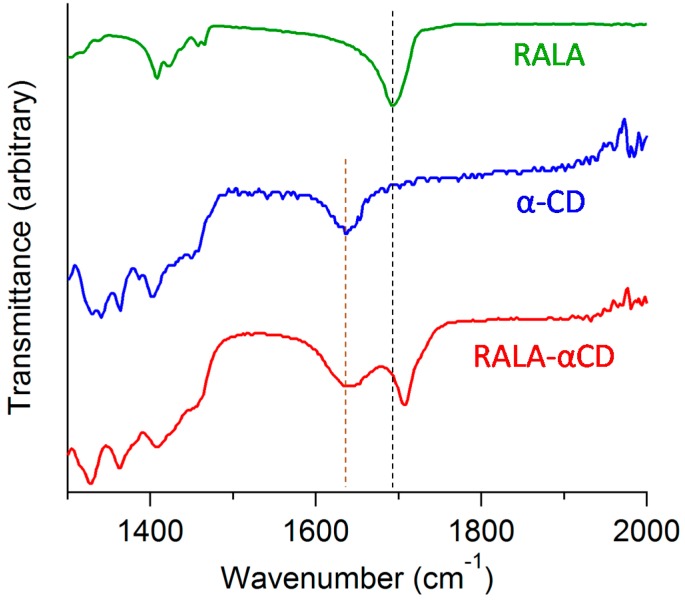
ATR/FT-IR spectra of the crystalline RALA-αCD complex, α-CD and RALA.

ATR/FT-IR spectroscopy can be used to analyze spectral signals and determine the existence of intermolecular interactions between the RALA and CD molecules. Microscopic FT-IR was used in our previous study to obtain a spectrum of the raw RALA-αCD complex [10]. In this study, ATR/FT-IR was applied to analyze the purified crystalline RALA-αCD complex, and the resulting spectrum is shown in Figure 4 for wavelengths in the range of 1300–2000 cm^−1^. The ATR/FT-IR spectra of RALA and α-CD are also shown in Figure 4 for comparison. The ATR/FT-IR spectra of crystalline RALA showed a peak at 1693 cm^−1^ due to the stretching of the carbonyl group in the carboxylic acid, which was in agreement with the results of our previous study [10]. Following the complexation of the RALA with α-CD, the ν_as_ (C=O, carbonyl group of carboxylic acid) of RALA shifted from 1693 to 1707 cm^−1^. This higher frequency shift of the carbonyl group can be explained considering the interruption of the strong hydrogen bonding existed in RALA following the formation of the RALA-αCD complex. This result indicated that the microscopic environment of the C=O group of RALA in the RALA-αCD complex had changed compared with that of free RALA and that the carbonyl group of the crystalline RALA-αCD complex existed in a more hydrophobic circumstances than that of free RALA itself.

### 2.5. Microscopic Raman Spectroscopy

**Figure 5:**
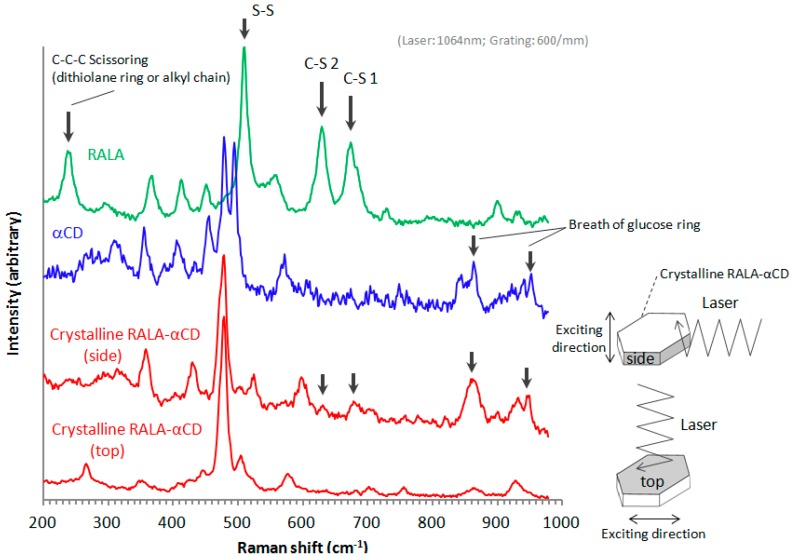
Raman spectra of the crystalline RALA-αCD complex, RALA and α-CD.

The purpose of this study was to explore the small changes in the 1,2-dithiolane ring of RALA using microscopic Raman spectroscopy. In a previous work, microscopic Raman spectroscopy using visible light, 633 nm He–Ne and 514.5 nm Ar^+^ laser, excitations successfully detected the effect of CD complexation on the structural properties of RALA in a previous study [10]. In this work, we used a near-infrared 1064 nm laser to obtain a clearer spectrum of the guest molecule than our previous work using visible light [10]. The Raman spectra of the crystalline RALA-αCD complex, α-CD and free RALA were recorded in the range of 200–1000 cm^−1^ and normalized (Figure 5). The Raman spectrum of free RALA contained a *ν*_1_ band due to S–S stretching around 510 cm^−1^ and *ν*_2_ bands due to two C–S stretching around 630 and 675 cm^−1^. The Raman spectra of the crystalline RALA-αCD complex (side and top) showed that the *ν*_1_ or *ν*_2_ mode of RALA was significantly weakened by the complexation process. This result suggested that the all vibrational modes of the S–S and two C–S bonds in RALA had become almost Raman inactive because of conformational changes resulting from the complexation process with α-CD. We previously reported similar spectral changes for non-crystalline RALA-αCD complexes [10].

In this study, we additionally found that the spectral change due to complex formation is crystallographically anisotropic. As shown in Figure 2, a single crystal of the RALA-αCD complex forms a thin hexagonal plate in shape. Most crystallographic studies on α-CD complexes have shown that the guest molecules generally exhibit crystallographic disorder [16] and that it can be difficult to fix the structures of these complexes, especially those of the guest molecules. For this reason, it is important to conduct spectroscopic measurements that focus specifically on the guest molecules in these complexes. In this study, we were aiming to get the spectroscopic information of the guest molecules in the crystalline RALA-αCD complex, which was purified and the molecules would be arranged in a regular pattern, and the Raman spectra of this material would be anisotropic. Based on this working hypothesis, we measured the Raman spectra of the crystalline RALA-αCD complex from different angles, *i.e*., on the top and side of the hexagonal crystals (see illustrates in Figure 5) under a microscope.

As shown in Figure 5, the molecules were excited vertically relative to the direction of the laser for these measurements. When the Raman spectrum of the crystalline RALA-α CD complex was measured from the side direction, the breath of the glucose ring of α-CD [17,18] appeared around 860 and 950 cm^−1^. However, when the Raman spectrum of the complex was measured from the top direction, the breath of the glucose ring was very weak compared with that of the spectrum recorded from the side direction. These results therefore indicated that the α-CD molecules were arranged as a hexagonal thin crystal with its primary or secondary face directed towards the top of the hexagonal crystal and its side face directed towards the side of the crystal. With regard to the main bands of the RALA molecules, the bands belonging to the S–S and C–S stretching vibrations appeared around 510 (S–S), 630 (C–S 2) and 675 (C–S 1) cm^−1^ also varied their signal intensities depending on the direction of the laser irradiation. When the laser was irradiated from the side direction of the crystalline RALA-αCD complex, the stretching vibrations of the two C–S bonds were observed nearby 630 and 679 cm^−1^ respectively. In contrast, the C–S bonds stretching vibrations were very weak and almost disappeared when the crystals of the complex were irradiated with laser light from the top direction. The peak around 480 cm^−1^ is the characteristic out-of plane deformation of the glucose ring of α-CD [17,18] and for both of the spectrum excited from the different directions, this band around 480 cm^−1^ was Raman active (Figure 5, red lines). The relative intensities of C–S peaks to the peaks around 480 cm^−1^ was clearly weaker when the laser was irradiated from the top compared from the side. These results suggested that the guest RALA molecule had been successfully encapsulated in the α-CD cavity with 1,2-dithiolane ring projecting in a vertical direction relative to the α-CD ring.

Another characteristic band of RALA appeared around 240 cm^−1^. This band was attributed to the scissoring or out-of-plane bending vibration of a C–C–C moiety belonging to the 1,2-dithiolane ring or alkyl chain of RALA, using the Gaussian-09 program package [19]. The C–C–C bending vibration (240 cm^−1^) also occurred for free RALA, but it was not detected in the case of the crystalline RALA-αCD complex. From these results, it was envisioned that the α-CD molecules in the crystalline RALA-αCD complex were arranged in a regular pattern, and that the Raman spectra of this material was anisotropic.

Barrientos *et al*. [20] reported the PXRD patterns of several other hexagonal crystal structures, including the complexes of α-CD (host) with 1-octylamine and 1-octanethiol (guests). These results suggested that the PXRD pattern of the crystalline RALA-αCD complex indicated that the RALA-αCD molecules were put the channel structures in order through two dimensional network interactions between the guest and the host (α-CD) molecules, with the α-CD network running parallel to the hexagonal faces. From the Raman spectrum of the RALA-αCD complex, it is clear that the 1,2-dithiolane ring of RALA was captured in the α-CD cavity and positioned perpendicular to the plane of α-CD ring. These results also show that the carboxylic acid group of RALA was exposed to the surface of the (001) hexagonal face but that it did not interact with α-CD, as indicated in the ATR/FT-IR spectra in Figure 4. These groups could potentially interact with another unit lattice through a self-assembly process.

### 2.6. Solid-State NMR Study

**Figure 6:**
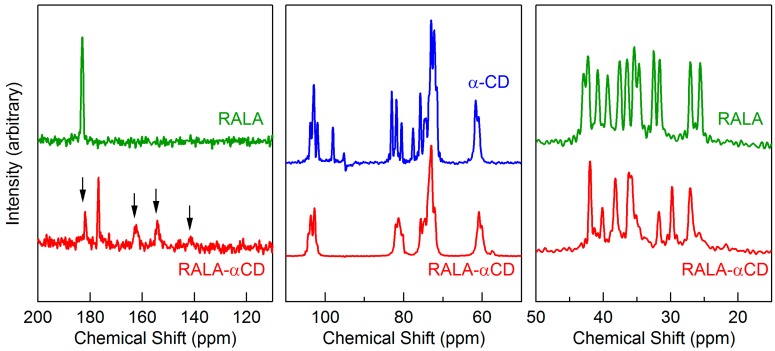
^13^C-CP/MAS NMR spectra of the crystalline RALA-αCD complex, free α-CD, and free RALA. The arrows indicate the spinning side bands.

A variety of different analytical methods have been developed to evaluate the conformational characteristics of compounds in the solid-state, including solid-state fluorescence spectroscopy, FTIR, and solid-state NMR [21]. ^13^C cross-polarization (CP)/magic angle spinning (MAS) NMR is a powerful analytical tool, which was used in this study for the crystallographic investigation of the RALA-αCD complex. In general, the ^13^C-CP/MAS NMR spectra of free CDs exhibit multiple resonance carbon atoms in each type. These features are explained to correlate with different torsion angles about the α(1→4) linkages, and the different torsion angles can be used to describe the difference of the orientation of hydroxyl groups [21]. For the crystalline RALA-αCD complex, the multiplicities of the different carbon atoms were reduced and the resulting spectrum was compared with that of free α-CD. The results revealed that the chemical shifts of the resonance signals of the crystalline complex were almost the same as those of free α-CD (see Figure 6, 50–110 ppm). The ^13^C-CP/MAS NMR spectrum of the crystalline RALA-αCD showed different spectral patterns from those of α-CD in the range of CD resonances (50–110 ppm) (Figure 6). Specifically, the multiplicity of signals for each carbon atom was reduced in the case of RALA-αCD. It is well known that the free CD spectrum exhibits multiple resonances for each type of carbon atom. These features have been responsible for the different torsion angles around the α(1→4) linkages and have been correlated with the torsion angles that described the orientation of the hydroxyl angles [22]. These results suggested that RALA and α-CD had formed an inclusion complex in the crystalline RALA-αCD complex and that the enhanced symmetry of the α-CD macrocycle had been attained in the crystalline complex compared with the free α-CD. Furthermore, consideration of the NMR spectra of free RALA of the crystalline RALA-αCD complex between 110 and 200 ppm (Figure 6), revealed that the peak observed around 180 ppm could be assigned to the C=O group of RALA. This peak appeared at 182.9 ppm for free RALA, but was shifted to a higher field (176.9 ppm) for the crystalline RALA-αCD complex. These results indicated that the microscopic environment of the C=O group of the RALA in the crystalline RALA-αCD complex had changed from that of free RALA.

Consideration of a higher magnetic field between 50 and 20 ppm revealed that there were multiple peaks in the range of 20–45 ppm for the RALA-αCD complex, which could be assigned to –CH bonds. Multiple resonance signals were also observed in the same region of the spectrum for free RALA. A single-crystal structure of lipoic acid was reported in the literature, which showed that the material existed as a dimer [23]. However, the ^13^C-CP/MAS NMR spectrum of the crystalline RALA-αCD complex prepared in the current study showed that the signal multiplicity was reduced compared with free RALA. For the crystalline RALA-αCD complex, the peaks in the range of 20–45 ppm indicated that structure of RALA had converged, which could also explain the shift in the C=O peak of the crystalline RALA-αCD complex. RALA itself exists as a dimer with the two RALA molecules interacting with each other through the formation of strong hydrogen bonds. In the case of the crystalline RALA-αCD complex, the hydrogen bonding interactions between the two RALA molecules would be broken in favor of the formation new interactions between the RALA and α-CD molecules, which would result in the observed shift in the C=O peak.

The results of the ^13^C-CP/MAS NMR analysis were in accordance with the ATR/FT-IR results. Taken together, these results show that the microscopic environment of the C=O group of free RALA changed following its complexation with α-CD. These results therefore indicated that the structure of the crystalline RALA-αCD complex prepared in the current study existed as one structure, meaning that it had a low level of disorder in its structure.

**Figure 7:**
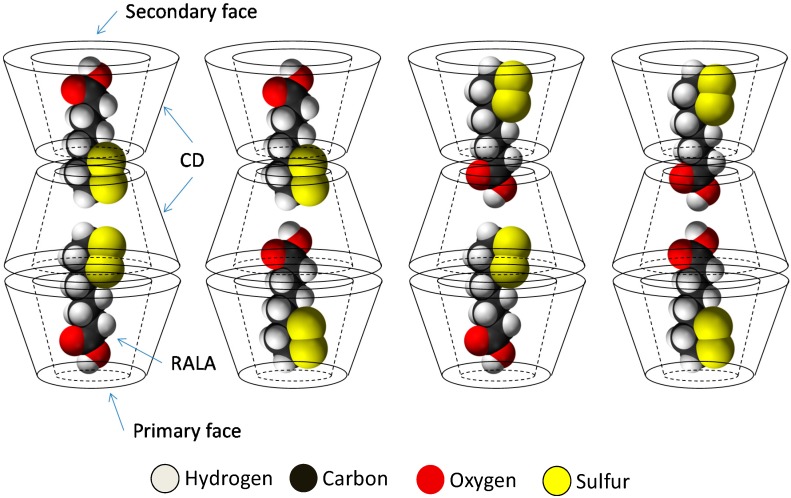
Sketch of the different topologies for the crystalline RALA-CD complex.

The results of PXRD and ^1^H-NMR show that the crystalline RALA-αCD complex is a channel type 2:3 (RALA:α-CD) complex and the results of ATR/FT-IR indicated that the carbonyl group of RALA in the crystalline RALA-αCD complex existed in a hydrophobic environment. The result of the microscopic Raman spectroscopy indicated that the α-CD molecules were arranged as a hexagonal thin crystal with its primary or secondary faces directed towards the top of the hexagonal crystal and its side face directed towards the side of the crystal. From the Raman spectrum of the RALA-αCD complex, it is clear that the 1,2-dithiolane ring of RALA was penetrated into the α-CD cavity and positioned perpendicular to the plane of α-CD ring. All these data and the ^13^C-CP/MAS NMR analysis can provide the possible topological structures for the crystalline RALA-αCD complex (Figure 7).

## 3. Experimental Section

### 3.1. Chemicals

*R*(+)-α-Lipoic acid sodium salt (NaRALA) was purchased from Toyo Hakko Co., Ltd. (Obu, Japan). CAVAMAX^®^ W6 FOOD (α-CD) was purchased from Wacker Chemie AG (München, Germany). The solvents used for spectrophotometry were purchased from Wako Pure Chemical Ind., Ltd. (Osaka, Japan). Deuterated dimethyl sulfoxide (DMSO-*d*_6_; NMR measurement grade) was purchased from Sigma-Aldrich Japan K.K. (Osaka, Japan). All of the reagents used in this study were purchased as analytical grade. The water used in the current study was generated using a Milli-Q^®^ water purification system (Merck Millipore, Billerica, MA, USA). All of the chemicals were used as supplied without further purification.

### 3.2. Equipment

The mechanical stirrer (EYELA NZ-1000) and the freeze dryer (EYELA FD-1000) units used in this study were supplied by Tokyo Rika Kikai (Tokyo, Japan). ^1^H-NMR measurements were conducted on a JNM ECA-600 spectrometer (JEOL Ltd., Tokyo, Japan). ^13^C-CP/MAS NMR measurements were conducted on a JNM ECA-300 spectrometer (JEOL Ltd.). Scanning electron microscopy (SEM) images were recorded on an S-4500 SEM system (Hitachi, Toyko, Japan). Particle size distribution data were analyzed using the Scandium software package (Olympus, Tokyo, Japan). Powder X-ray diffraction (PXRD) patterns were measured with an Ultima IV XRD system (Rigaku Corporation, Tokyo, Japan). Attenuated total reflectance (ATR) spectra were recorded on a Nicolet iS 10 MX FT-IR spectrometer (Thermo Fisher Scientific, Waltham, MA, USA). Microscopic-Raman spectra were recorded using a Raman system with LabRAM HR800 spectrometer (HORIBA Jobin Yvon, Palaiseau, France). Solid-state NMR analysis was conducted on an ECA-300 spectrometer (JEOL Ltd., Tokyo, Japan).

### 3.3. Preparation of the Crystalline RALA-αCD Complex

The powdered RALA-αCD complexes were prepared according to our previously published procedure [11] with molar ratios of 1:2 and 2:3 (RALA:CD). The corresponding crystalline RALA-αCD complexes were prepared by mixing the RALA-αCD powders with distilled water. The resulting mixtures were heated at 90 °C to give a supersaturated solutions, which were subsequently filtered and spontaneously cooled to room temperature. The resulting precipitates were collected by filtration and dried at room temperature.

### 3.4. ^1^H-NMR

RALA, α-CD and the crystalline RALA-αCD complexes were separately dissolved in DMSO-*d*_6_, and the resulting solutions were analyzed by one-dimensional ^1^H NMR (^1^H-NMR). The ^1^H-NMR spectra were recorded at room temperature on a JNM ECA-600 spectrometer (JEOL Ltd.). The chemical shifts (δ) have been reported in ppm relative to tetramethylsilane (δ = 0 ppm).

### 3.5. Morphological Characterization by Scanning Electron Microscopy (SEM)

SEM images were collected by sprinkling a sample of each RALA-αCD complex onto a palladium SEM stub covered in conductive glue. The stubs were then sputter coated with gold for 3 min before being measured at 15 kV using an S-4500 SEM system for morphological analysis. Three different fields within each sample were randomly chosen and three images from each field were taken at magnifications of 300×, 500×, 1000×, and 10,000× to give a total number of 12 images per sample.

### 3.6. Powder X-ray Diffraction (PXRD) Measurements

PXRD measurements were recorded on Ultima IV system using graphite-monochromated Cu Κα radiation (λ = 1.54178 Å) at 60 kV and 150 mA. Measurements were obtained using a scanning interval of 2θ between 3° and 50° with a scanning speed of 2 °/min.

### 3.7. ATR/Fourier Transform Infrared (FT-IR) Spectroscopy

ATR spectra were recorded on a Nicolet iS 10 MX FT-IR spectrometer. The powdered RALA-αCD complexes were placed on an equipped stage and the data were recorded in the range of 400–4000 cm^−1^ in the total reflection mode with a spectral resolution of 4 cm^−1^.

### 3.8. Microscopic-Raman Spectroscopy

Microscopic-Raman spectra were collected on a micro-Raman system equipped with an 800 mm single spectrometer and a BX41 optical microscope (Olympus, Tokyo, Japan). In this study, we used a 1064 nm Nd-YAG laser that was focused on the crystalline samples using a 100× objective lens (Leica, HCX PL FLUOTAR, NA = 0.75). Scattered light was collected in a backscattered geometry. The edge filter, pinhole (1000 μm in diameter) and slit (100 μm in width) were properly arranged to gain high-resolution spectra from the microcrystals. All of the spectra collected in the current study were obtained in the spectral range of 100–900 cm^-1^ with pixel resolutions of 1.4–1.7 cm^−1^ for a 300 groove/mm grating and a 512-pixel InGaAs detector.

### 3.9. Solid-State NMR Study

^13^C-CP/MAS NMR measurements with a π/2 pulse (2.5 μs) and high-powered decoupling using two pulse phase-modulated decoupling (phase modulation angle of 15 degrees) were recorded on an ECA-300 spectrometer with a frequency of 74.2 MHz for ^13^C. The spectrometer was equipped with a 4.0 mmϕ MAS probehead. The repetition delay time and contact time were set as 3 s and 1 ms, respectively. All of the measurements were performed at 6 kHz of the MAS spectra, and 16,384 free induction decays were collected and averaged to obtain the ^13^C spectra of the complexes. Adamantane was used as an external standard (29.5 ppm).

## 4. Conclusions

The purpose of this study was to develop a better understanding of the host-guest interactions of RALA-αCD complexes and evaluate a crystalline RALA-αCD complex using ATR/FT-IR, micro-Raman spectroscopy and ^13^C-CP/MAS NMR. ATR/FT-IR analysis showed that the C=O stretching vibration due to the carboxylic group of RALA observed at 1693 cm^–1^ and shifted to 1707 cm^−1^ (RALA-αCD) following the complexation of RALA with α-CD. These results indicated that the C=O group of RALA existed in a hydrophobic circumstances by the formation of the RALA-αCD complex. This observation represents a new finding over the results of our previous study for non-purified RALA-αCD, where it was not possible to evaluate the interaction of the C=O group of RALA with α-CD. Micro-Raman spectroscopy showed that the S–S and C–S stretching vibrations, which were observed at 510 (S–S), 630 (C–S 2) and 675 (C–S 1) cm^−1^ for non-complexed free RALA were weakened significantly (almost disappeared) by the formation of the crystalline RALA-αCD complex. Furthermore, the C–S vibration varied considerably depending on the direction of the excitation laser. When the laser was irradiated from the side of the crystalline RALA-αCD complex, the stretching vibrations of the C–S bonds were observed around 630 and 679 cm^−1^. However, when the laser was irradiated from the top of the crystal, the stretching vibrations of C–S bonds were very weak. These results indicated that the conformation of the 1,2-dithiolane ring of RALA had changed after the formation of the RALA-αCD complex. These results also showed that the 1,2-dithiolane ring of RALA had entered the cavity of α-CD and that it was perpendicular to the plane of the α-CD ring. Solid-state NMR analysis indicated that the α-CD formed a complex with RALA because the chemical shift of the C=O group in RALA was shifted to a higher magnetic field for the crystalline RALA-αCD complex. The results of the ^13^C-CP/MAS NMR analysis of the RALA-αCD complex were in accordance with the results obtained from the ATR/FT-IR measurements. The microscopic environment of the C=O group of free RALA changed following the formation of the RALA-αCD complex, where the carbonyl group existed in a more hydrophobic circumstances compared with free RALA.

The remarkable shifts in the vibrational modes of the ATR/FT-IR, Raman, and ^13^C-CP/MAS NMR spectra confirm the formation of a crystalline RALA-αCD complex and provide valuable information pertaining to the structure of this complex. These spectra also revealed that several peaks for O–H vibrations had shifted or changed in their intensity. Taken together, these results indicate that α-CD and RALA form a host-guest inclusion complex. Furthermore, the results of PXRD and ^1^H-NMR show that the crystalline RALA-αCD complex is a channel type 2:3 (RALA:α-CD) complex. Barrientos and co-workers [20] synthesized α-CD/C_8_H_17_SH (1-octanethiol) and 2α-CD/C_8_H_17_NH_2_ (octylamine) complexes and evaluated their physic-chemical properties using PXRD, SEM imaging, and Raman spectroscopy. They showed that 2α-CD/C_8_H_17_NH_2_ crystals had hexagonal shape and in the crystal structure, the supramolecular complexes were put in the channel structures in order. Additionally, they showed a schematic representation of the corresponding inclusion complexes. In this study, the crystalline complex made of RALA (one of the octanoic derivatives) and αCD also showed hexagonal channel-type structure. The results of this study therefore indicate that there are four possible topological structures for the crystalline RALA-αCD complex (Figure 7).
